# The Planorbid Snail *Biomphalaria glabrata* Expresses a Hemocyanin-Like Sequence in the Albumen Gland

**DOI:** 10.1371/journal.pone.0168665

**Published:** 2016-12-30

**Authors:** Janeth J. Peña, Coen M. Adema

**Affiliations:** Center for Evolutionary and Theoretical Immunology, Department of Biology, University of New Mexico, MSCO3 2020, Albuquerque, NM, United States of America; George Washington University School of Medicine and Health Sciences, UNITED STATES

## Abstract

The parasitic flatworm *Schistosoma mansoni*, causative agent of human intestinal schistosomiasis in South America, relies importantly on the freshwater snail *Biomphalaria glabrata* as intermediate host to achieve development of cercariae that infect humans. The recommendation from the World Health Organization (WHO) to integrate snail control in efforts to counter schistosomiasis transmission provides impetus for in depth study of *B*. *glabrata* biology. Our analysis indicates that two distinct hemocyanin-like genes (*hcl-1* and *hcl-2*) are present in *B*. *glabrata*, a snail that uses hemoglobin for oxygen transport. Characterization of BAC clones yielded the full length *hcl-1* gene, which is comprised of three functional unit (FU) domains at the amino acid level. Database searches and *in silico* analyses identified the second *hcl* gene (*hcl-2*), composed of six FU domains. Both genes are unusual for lacking canonical residues and having fewer FU domains than typical molluscan hemocyanins that contain 7–8 FUs. Reverse transcription PCR demonstrated that Hcl-1 is expressed in a manner that correlates with reproductive maturity in the albumen gland (AG), an immune- and reproduction-relevant organ. Immune cross-reactivity with anti-keyhole limpet hemocyanin (α-KLH) antiserum and tandem-mass spectrometry validated the presence of Hcl-1 protein in the AG and egg mass fluid (EMF). The evolutionary conservation of hemocyanin-like sequences in *B*. *glabrata* in the presence of the oxygen carrier hemoglobin, combined with our results, suggest that the Hcl-1protein has a functional role in general and/or reproductive biology. Further investigations are needed to explore Hcl-1 as a potential target for snail control.

## Introduction

Human schistosomiasis is caused by parasitic blood flukes of the genus *Schistosoma* that utilize freshwater snails as intermediate hosts for development and asexual reproduction to progress to cercariae, the human infective stage. This parasitic disease afflicts 208 million people worldwide and leaves 600 million at risk in endemic areas [1]. *Schistosoma mansoni*, the causative agent of intestinal schistosomiasis in endemic regions of South America, relies importantly on the planorbid snail *Biomphalaria glabrata* as a major snail intermediate host for transmission of infection to humans [2] and related *Biomphalaria* species transmit *S*. *mansoni* in Sub-Saharan Africa [3]. The World Health Organization (WHO) recommends integrated control of both parasite and snail intermediate host to reduce disease transmission [4]. In absence of available vaccines [5], current approaches include mass-drug administration (MDA) [6], management of snail habitats [7], use of molluscicides [8, 9], biological control of snails [10, 11, 12] and public health education [13]. Unfortunately, MDA does not protect against re-infection, hampering long-term control of schistosomiasis in parasite-endemic regions [14]. Negative ecological impact may limit general application of non-specific molluscicides [15].

Long-term, sustainable disease control requires further knowledge of biology of the snail *B*. *glabrata* in order to develop and integrate alternative control methods to counter the intermediate host and thereby transmission of schistosomiasis. *Biomphalaria glabrata* belongs to the family Planorbidae; ramshorn snails that use iron-containing hemoglobin to transport oxygen through their hemolymph, giving them red pigmented blood [16]. This is unusual since other gastropods (basal taxa and sister families) do not have hemoglobin but employ hemocyanin, a blue-pigmented protein that uses copper to bind oxygen for transport [17]. It was postulated that *B*. *glabrata* hemoglobin has evolved through gene duplications and fusion involving myoglobin, a protein crucial in oxygenation of muscle tissue [16]. Although hemocyanin is an efficient oxygen carrier [18]; hemoglobin is thought to possibly provide planorbid snails a greater diving capacity that may offer an evolutionary advantage over other snails that employ hemocyanin [19]. The advent of hemoglobin has not expunged all traces of hemocyanin; partial hemocyanin-like EST sequences have been recorded from *B*. *glabrata* and a hemocyanin-like protein ultrastructure was visualized from the hemolymph [16], and in mantle rhogocytes [20]. Hathaway et al. [21], using mass spectrometry, detected the presence of hemocyanin-like peptides in the albumen gland (AG) and egg mass fluid (EMF) of *B*. *glabrata*. A microarray study indicated increased expression of hemocyanin-like transcripts in immune-challenged *B*. *glabrata* [22]**.** The above raises a question regarding the origin and functional significance of hemocyanin-like sequences by *B*. *glabrata* in the presence of hemoglobin.

Molluscan hemocyanins belong to the copper type 3 protein superfamily. The functional unit (FU) domains of copper type 3 proteins have an active site that contains six canonical histidines that bind two copper atoms. Generally, molluscan hemocyanins are composed of monomers that have 7–8 FU domains that each have unique sequence features and are categorized by letters A-H [17]. Ten monomeric subunits multimerize to form a functional didecamer hemocyanin protein. The members of the copper type 3 protein superfamily, that also includes single-domain enzymes such as phenoloxidases and tyrosinases, may have one of a variety of biological functions ranging from pigment formation, innate immunity, reproduction and, in the case of hemocyanin, oxygen transport [23–26]. Some molluscan and crustacean hemocyanins, which resulted independently from different subclasses of copper type 3 proteins through convergent evolution, are reported to have phenoloxidase activity *in vitro*[27–29], allowing them to produce melanin, an important compound involved in immunity of invertebrates[30] and in pigmentation [31].

This study explores the origins of hemocyanin-like sequences in *B*. *glabrata* and identifies two different hemocyanin-like genes (*hcl-1* and *hcl-2*). *Biomphalaria glabrata hcl-1* is characterized further because its presence in both the AG and EMF indicates a putative involvement in reproduction, and/or immunity and reveals insights into gastropod biology.

## Materials and Methods

### Snails

*Biomphalaria glabrata* snails of the M line and BB02 strains and of two field isolates, referred to as VG2 and VG3, are maintained at the University of New Mexico [32]. Snails were housed in tanks containing artificial spring water [33] at 26°C and fed red leaf lettuce *ad libitum* and chicken pellets weekly. VG2 and VG3 were collected in 2009 from Virgem das Graças, Minas Gerais, Brazil, and identified as *B*. *glabrata* based on 16S, CO1 and ND1 sequences (JQ886405-10). The BB02 strain has been used to characterize the *B*. *glabrata* genome (VectorBase; www.vectorbase.org; assembly BglaB1).

### Full length sequencing and computational analysis of *hcl-1*

Expressed sequence tags (CN779709, CN779680, CN655026, CN549169) obtained from M line *B*. *glabrata* [16] containing hemocyanin-like sequences were used to screen the BBO2 genomic BG_BBa BAC library [34]. Of several positive BAC clones, two contiguous inserts (BG_BBa50I14 and BG_BBa28B18) were sequenced to obtain a full length hemocyanin-like gene (*hcl-1*). Sequencing was performed in house at the Molecular Biology Facility at the University of New Mexico and commercially at The Genome Institute at Washington University, Saint Louis, MO. Sequence reads were assembled using Sequencher 5.1 (GC Codes). BAC insert sequences were submitted to GenBank (KU682269, KU682270). Open reading frame and BLAST analyses were used for *in silico* prediction of intron/exon splice junctions, also considering canonical CAG/GT motifs. The *hcl-1* gene sequence was screened to identify the 5’UTR, Kozak sequence, start codon, stop codon and polyadenylation signal (AATAA). SignalP V 4.1 [35] was used for signal peptide prediction. The *in silico* assembled and annotated sequence was used to design primers for experimental confirmation with RT-PCR of cDNA sequence in BB02, M line, VG2 and VG3 strains of *B*. *glabrata*. Protein mass was predicted for Hcl-1 with the software Compute pI/Mw tool [36] for protein characterization. Public databases (GenBank and VectorBase) were also scanned with Hcl-1 for presence of additional hemocyanin-like proteins. Copper type 3 proteins from *B*. *glabrata* were aligned (ClustalW; http://www.genome.jp/tools/clustalw/) with additional gastropod hemocyanins and a tyrosinase from a bivalve (See legend Fig 3 for accession numbers). A gene-tree was generated using MEGA 6 [37], (Neighbor-joining) using default parameters and the software suggested model for evolution (Poisson correction method).

### RNA extraction

For various experiments, RNA was extracted from adult *B*. *glabrata* snails (10–15 mm shell diameter), juveniles (4–9 mm), pooled newly hatched snails (within 2 days of emergence), and pooled embryos from whole egg masses. The shell was removed from individual snails to obtain whole body tissues, with exception of the newly hatched snails. RNA from snail embryos was extracted from whole egg masses. Samples were disrupted using a plastic pestle (Kontes RNase-free) in 1 mL of Trizol-Reagent (Invitrogen) and processed according to manufacturer’s instructions. The RNA was treated with TURBO DNA-free (Ambion) to remove residual genomic DNA. The RNA was quantified spectrophotometrically using the NanoDrop 2000c (Thermo Scientific). Quality was checked using 2100 Bioanalyzer with the RNA Nano Kit (Agilent).

### Reverse transcription-PCR and sequencing

RNA from individual BB02, M-line, VG2 and VG3 snails was reverse transcribed to cDNA (Omniscript RT Kit, Qiagen), using 2 μg of RNA per reaction, with an oligo (dT) primer. AmpliTaq Gold (Applied Biosystems, Life Technologies) was used for PCR, employing Hcl-1 primers that span introns, designed from the *in silico* transcript prediction (S1 Table) and 1μL of RT-reaction. The *B*. *glabrata* eukaryotic translation initiation factor eIF2α (GenBank KF648316) (Primers; F- CCTGCAGGTTCTACAAAACA; R-TATCCTTACCTTTATCTTTGTCTACTC) was used as positive control. Negative controls consisted of omitting the reverse transcription step, and using water instead of template. The cycling conditions were as follows: 95°C for 10 min for initial denaturation; 25 cycles at: 95°C for 60 s, 30 s at annealing temperature (depending on primers used), 72°C for 60 s; then 72°C for 7 min for final extension, ramping rate set at 1°/s. RT-PCR amplicons were purified (QIAquick PCR Purification Kit, Qiagen) and sequenced (BigDye® Direct Cycle Sequencing Kit v3.1, Applied Biosystems, Life Technologies) using amplicon-specific primers. Extension products were read using a ABI 3100 sequencer. Sequences were edited by eye and aligned using Sequencher 5.1(GC Codes).

### Tissue specific expression of Hcl-1

The following tissues were dissected from an adult (14 mm shell diameter) M line snail; albumen gland, mantle, stomach, hepatopancreas, and ovotestis. RNA was extracted using Trizol-Reagent (Invitrogen, Carlsbad, CA). RNA quality checks and RT-PCR were performed as above, using Hcl-1 specific primers for a 440 bp amplicon with eIF2α as positive control. Amplicon presence was confirmed using 1% agarose gels stained with GelRed (Biotium). Two replicate experiments were performed with independent tissue samples.

### Hcl-1 expression during ontogeny

RNA was extracted from M line snails at different developmental stages; newly laid egg masses (48hrs), embryos with shell (96 hrs); newly hatched snails (≤2 day), and snails of 4, 8, 9, 10, 11, 12, and 15 mm in shell diameter. To investigate expression, RT-PCR with Hcl-1 specific primers targeting a 757 bp sequence fragment were used, with eIF2α as positive control. Detection of amplicons was performed as mentioned above.

### Immunodetection and mass spectrometry

The shell of an adult M line snail was crushed between glass slides and 10 μL of hemolymph were collected and centrifuged to remove cells (5 min, 15000g). The hemolymph supernatant was placed in Laemmli sample buffer (Bio-Rad) with increased SDS content of 20% (w/v), boiled for 5 minutes and kept on ice until use. The AG was dissected from adult M line snails (14–15 mm diameter), and individually disrupted with a pestle in 100 μl of Laemmli sample buffer with an increased SDS content of 20% (w/v) [21], and boiled for 5 minutes. The perivitteline fluid surrounding the embryos, referred to as egg mass fluid (EMF), was collected from snail eggs and processed according to Hathaway et al. [21]. All samples were spun to remove debris prior to gel electrophoresis.

The protein components of AG, EMF, and hemolymph were separated by SDS-PAGE (Mini-Protean II system, Bio-Rad). The 1.0 mm thick gel consisted of a 4% acrylamide stacking gel (0.1% SDS in 0.125 M Tris-HCl pH 6.8) and a 5–20% gradient acrylamide separating gel (0.1% SDS in 0.375 M Tris-HCl pH 8.8) [32]. To increase resolution of large proteins, electrophoresis was continued until the 50kDa marker band (Precision Plus Protein Kaleidoscope, Bio-Rad) ran off the gels (~3 hours). Protein profiles were transferred to a nitrocellulose membrane using the Trans-Blot SD Semi-dry system (Biorad). The membrane was incubated in blocking solution (0.1% Tween 20 in 20mM Tris pH 7.5, 150mM Nacl, 5% w/v nonfat dry milk) for 1 hr, washed 3X in TBS and incubated with polyclonal α-KLH (anti-Keyhole Limpet hemocyanin; Sigma H0892) at a 1:1000 dilution overnight at 4°C. The membrane was washed and treated with AP-conjugated goat α-rabbit as secondary antibody to detect antiserum reactive protein bands with NBT/BCPI as substrate. Alternatively, SDS-PAGE gels were rinsed with DI water, stained in Coomassie blue G250 (BioRad) and photo-documented.

For mass spectrometry, Coomassie blue stained bands of 150 kDa in the AG and EMF were excised from gel, destained, and digested with trypsin, for details see [38]. Peptide mass fingerprints (PMFs) were obtained on a matrix assisted laser desorption ionization tandem time-of-flight (MALDITOF-TOF) mass spectrometer (Ultraflex II; Bruker Daltonics Inc., Billerica, MA, USA). Porcine trypsin was used for internal mass calibration. Abundant peptides were subjected to tandem MS (MS/MS) to obtain information about the β- and γ-ions of the peptide sequence with a default setting of a precursor-ion mass tolerance of 0.6 Da recommended for MASCOT [38]. To identify proteins, we used Mascot (version 2.2; Matrix Science Inc., Boston, MA, USA) and combined PMFs and MS/MS in a search against the protein sequences of Hcl-1, Hcl-2 and proteins predicted from six frame translation of all *B*. *glabrata* ESTs in dbEST (NCBI). Our search allowed one missed cleavage during trypsin digestion. The molecular weight search (MOWSE) scores that indicated significant hits (p<0.05) were 21 for EMF and 26 for AG. No other peptide sequences yielded significant hits against *B*. *glabrata*. After the above approach yielded positive identification of Hcl-1, the default precursor–ion tolerance was relaxed to 1 Da to investigate whether additional PMF -recovered peptide masses matched *in silico* predicted tryptic fragments of Hcl-1 (PeptideMass software, [39]).

## Results

### *hcl-1* and *hcl-2* gene structure

**Fig 1:**
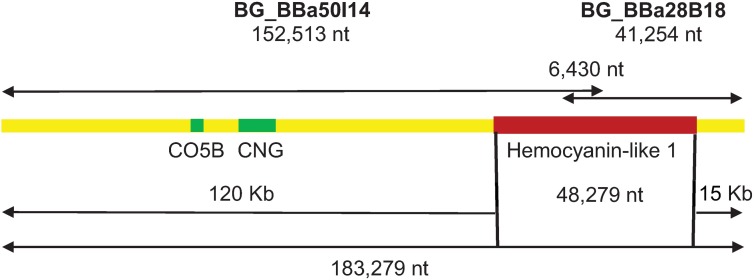
Genomic sequencing of *hcl-1*. Two contiguous BACs (BG_BBa 50I14 and BG_BBa 28B18) yielded a complete hemocyanin-like 1 gene (*hcl-1*). Additional genes were identified upstream of *hcl-1* with BLAST similarity to CO5B (cytochrome oxidase subunit 5B) and CNG (cyclic nucleotide-gated olfactory channel-like isoform).

**Fig 2:**
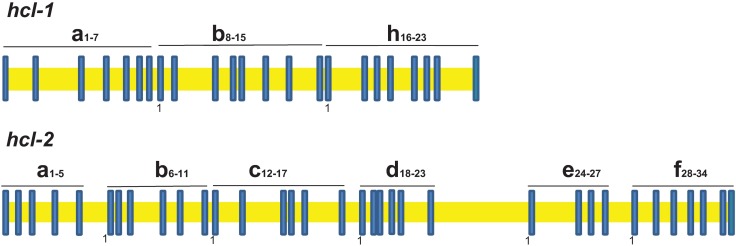
Intron-exon structure of hemocyanin-like genes of *B*. *glabrata*. The letters a-h designate the groups of exons that combine to encode a particular FU. The first exon of each FU is denoted with a one to indicate the conserved phase one intron upstream of the exons encoding the linker sequences that connect FUs. The FUs were annotated through comparisons with other gastropod hemocyanins.

A *B*. *glabrata* hemocyanin-like gene *(hcl-1*) encoding three FUs that were separated by two characteristic linker sequences was identified by sequencing of *B*. *glabrata* BB02 BAC clones (KU682269 and KU682270; Fig 1). Computational analysis revealed a gene structure consisting of 23 exons. The predicted coding sequence was experimentally obtained, yielding a cDNA transcript of 3981 nt (KU682271). The *hcl-1* gene model was annotated and is available in VectorBase (LGUN_ random _ scaffold 490; BGLB000218_RA). Inspection of predicted splice sites showed that intron phases were variable throughout the gene (Fig 2; S2 Table) and identified 11 non-consensus splice sequences (other than CAG), at the 3’ termini of introns. As is typical for molluscan hemocyanin gene sequences [40], the linker sequences in the *hcl-1* gene are preceded by phase 1 introns (interrupted after the 1^st^ nucleotide of a codon; Fig 2; S2 Table).

BLAST searches with the predicted amino acid sequence of Hcl-1 identified a second hemocyanin-like gene (denoted *hcl-2*) in *B*. *glabrata*. The *hcl-2* gene (~75000 nt) was reconstructed and annotated using genomic and RNA-seq data from the *B*. *glabrata* genome (NCBI Taxonomy ID 6526, and VectorBase assembly; LGUN_random_scaffold1005 28349–90182)**.** Analysis of *hcl-2* gene structure revealed 34 exons, yielding a predicted transcript of 6,027 nt, corresponding to six FUs, separated by linker sequences. The exons encoding linker sequences in the *hcl-2* gene are also preceded by phase one introns (Fig 2; S3 Table), similar to *hcl-1*.

### Hcl-1 and Hcl-2 are more similar to molluscan hemocyanins than to tyrosinases

**Fig 3:**
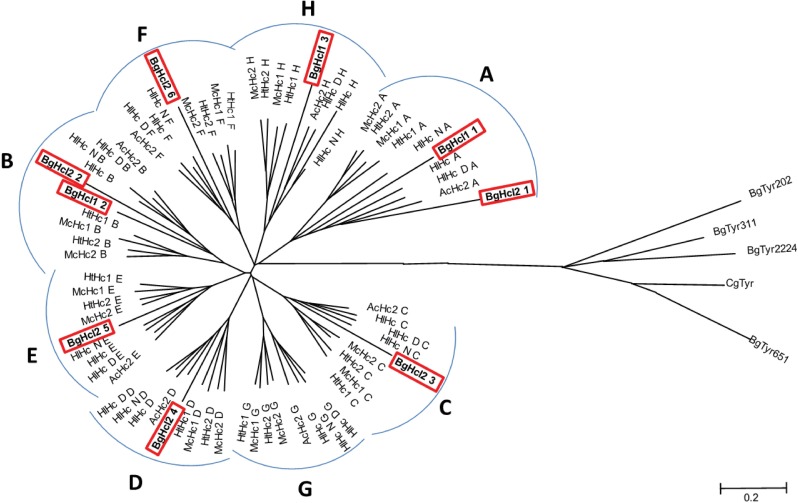
Identification of categories of functional unit domains by phylogenetic reconstruction. Generally, molluscan hemocyanins are composed of monomers that have 7–8 FU domains that each have unique sequence features and are categorized by letters A-H [17]. To better understand the structures of the Hcl proteins of *B*. *glabrata* and their relationship to other copper type 3 proteins, a phylogenetic comparison of FU domains was performed to infer the categories of FU domains present in Hcl-1 and Hcl-2. The evolutionary distances were computed using the Poisson correction method and are in the units of the number of amino acid substitutions per site. The analysis involved 73 amino acid sequences of FU domains from hemocyanins of 5 different gastropods; 2 vetigastropods (*Megathura crenulata* hemocyanin, McHc1 [CAG28309], McHc2 [CAG28308]; *Haliotis tuberculata* hemocyanin, HtHc1 [CAC20588], HtHc2 [CAC82192]), 3 heterobranchia (*Helix lucorum* hemocyanin, HlHc [AEO51766], HlHcD [AEO51767], HlHcN [AEO51768]; *Aplysia californica* hemocyanin, AcHc [CAD88977]; *B*. *glabrata* hemocyanin-like, BgHcl-1, BgHcl-2. Four *B*. *glabrata* predicted tyrosinase sequences (Tyr202 [XP_013065892], Tyr311 [XP_013078946], Tyr651 [XP_013065864], Tyr2224 [XP_013080599]) and a tyrosinase from the bivalve *Crassostria gigas* (CaTyr [XP_011420307]) were used for comparison against FU sequences from non-hemocyanin copper type 3 proteins. ClustalW (http://www.genome.jp/tools/clustalw/) was used for initial alignment; alignment screening and construction of the neighbor-joining tree were done in MEGA6 [37].

Scanning of the *B*. *glabrata* genome assembly using Hcl-1 amino acid sequence revealed additional hits for putative copper type 3 proteins. Four complete gene sequences, each consisting of a single copper type 3 domain were described (by automated annotation) as putative *B*. *glabrata* tyrosinases (S1 Fig). Phylogenetic comparison of FU domain sequences of all these copper type 3 proteins indicated that Hcl proteins group with molluscan hemocyanins and identified FU domains A, B, H in Hcl-1 and A-F for Hcl-2. Both Hcl proteins grouped separately from the putative molluscan single-domain tyrosinases (Fig 3).

### Amino acid sequences for Hcl-1 and Hcl-2

**Fig 4:**
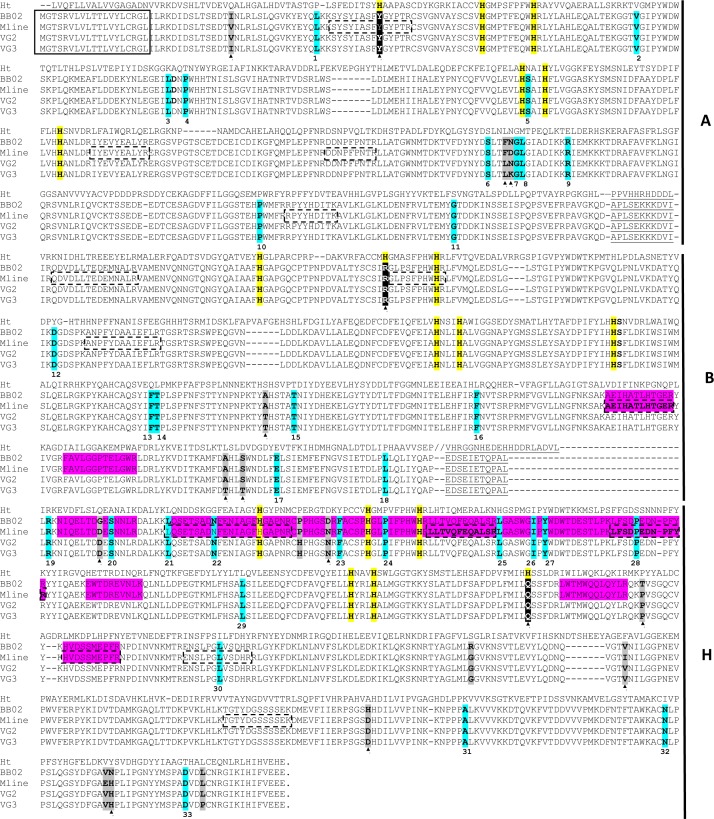
Alignment of Hcl-1 from four *B*. *glabrata* strains to the functional hemocyanin of *H*. *tuberculata*. The alignment includes Hcl-1 sequences of two lab strains (BB02 and M line) and two wild type snails (VG2 and VG3). Canonical histidines are highlighted in yellow, conserved replacements in those sites are highlighted in black. The amino acid linkers separating the functional units are underlined in all sequences. The signal peptide of Hcl-1 is boxed (note: the *H*. *tuberculata* hemocyanin gene [CAC20588] does not include a methionine start); grey highlights additional amino acid differences among *B*. *glabrata* strains; conserved replacements (relative to BB02) are denoted by the black triangles. Synonymous mutations are highlighted in blue; the numbers below the aligned sequences refer to details of specific mutations indicated in S4 Table (S4 Table). The peptide sequences matching to peptide masses detected by PMF analysis are boxed with a dashed line. Bold peptide sequences highlight the three peptides recovered by tandem MS (MS/MS). S5 Table (S5 Table) shows the protein sample (AG or EMF) of origin of the recorded peptides mass data. Peptides identified by MS analysis of 150 kDa proteins from AG and EMF in a previous study [21] that match the Hcl-1 sequence from *B*. *glabrata* snails are highlighted in pink.

The amino acid sequences for Hcl-1 and Hcl-2 are unusual compared to other molluscan hemocyanins. Generally, molluscan hemocyanins have seven or eight FU domains denoted A-H and each FU contains six canonical histidines that are required to form the copper-binding motif. Hcl-l has three FU domains classified as A, B, and H (Figs 2–4); each FU contains a conserved amino acid replacement of a canonical histidine. Hcl-2 consists of six FU domains (A-F; Figs 2 and 3), of which four FUs have one or more replacements of canonical histidines. The terminal domain F is incomplete, representing only half of a typical molluscan FU.

Experimentally obtained cDNA sequences confirmed that four different *B*. *glabrata* strains all have identical histidine replacements in Hcl-1. Using the BB02 Hcl-1 sequence as a reference, we identified additional differences in non-canonical amino acids among strains; six replacements occur in M line, six in VG2 and seven in VG3 (Fig 4).

### Hcl-1 is expressed in the albumen gland of *B*. *glabrata* in an age-dependent manner

**Fig 5:**
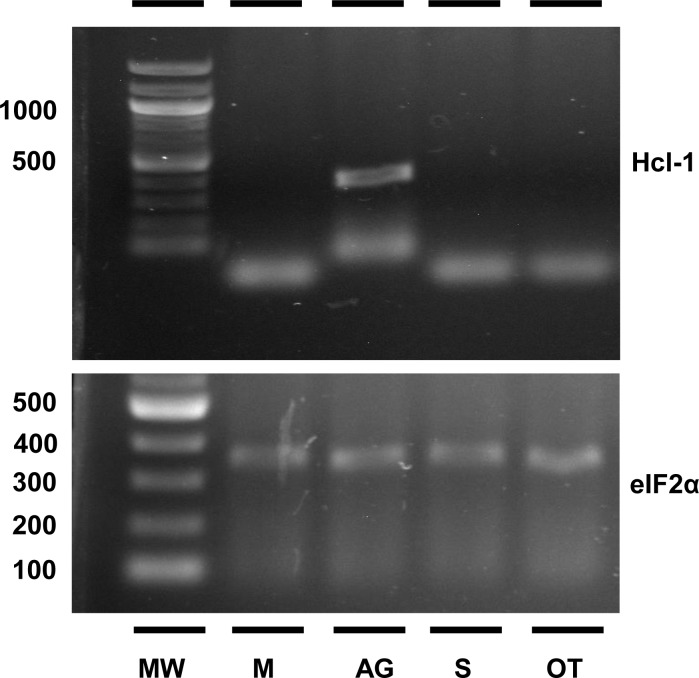
Hcl-1 is expressed in the albumen gland. Amplicons of 440 bp generated by RT-PCR using specific primers, (21F: TTGACGTTACTGACGCCATGA, 23R: AAGTCAAGTTTGTTACCGACGACG), demonstrate that Hcl-1 is only expressed in the AG. For positive control, eIF2α was used. MW: molecular weight, M: mantle, AG: albumen gland, S: stomach, and OT: ovotestis.

**Fig 6:**
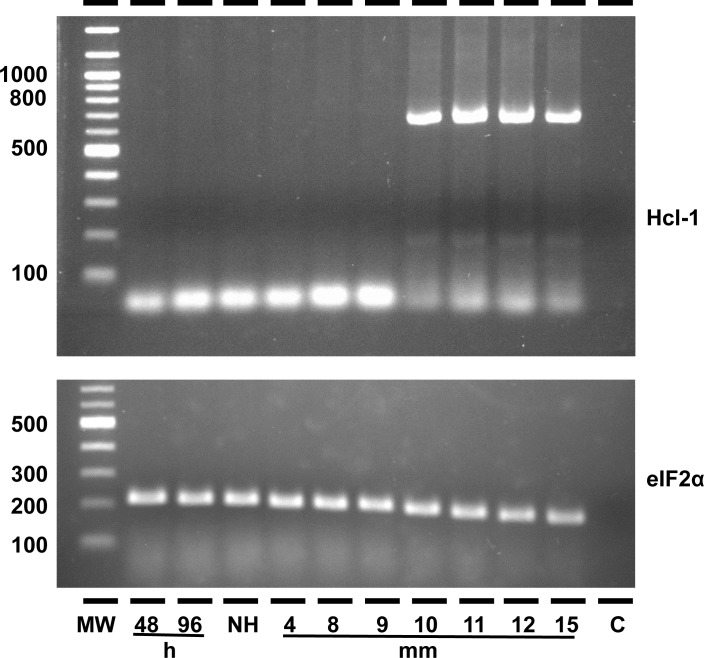
Hcl-1 is expressed in reproductively mature snails. Hcl-1 is only expressed in snails with a size of ≥10 mm. RT-PCR using Hcl-1 specific primers that span four introns (9F: TGATCCAATCTTTTACATTCATCAC, 13R: AAGTTGTCAGCGCTAGTCTCA) yielded an expected amplicon of 757 bp. eIF2α was used as positive control. MW: molecular weight, 48hrs, 96hrs, Nh: Newly hatched, 4, 8, 9, 10, 11, 12 and 15mm. C: water as negative control.

Among the tissues tested, Hcl-1 mRNA was only expressed in the albumen gland (Fig 5). This correlates with Hathaway et al [21], who detected hemocyanin-like peptides in the AG and EMF of *B*. *glabrata*. Hcl-1 is expressed in an age-dependent manner, commencing only after *B*. *glabrata* snails attain a size of 10 mm or greater (Fig 6), which is associated with reproductively mature snails.

### Hcl-1 protein is present in the albumen gland (AG) and egg mass fluid (EMF)

**Fig 7:**
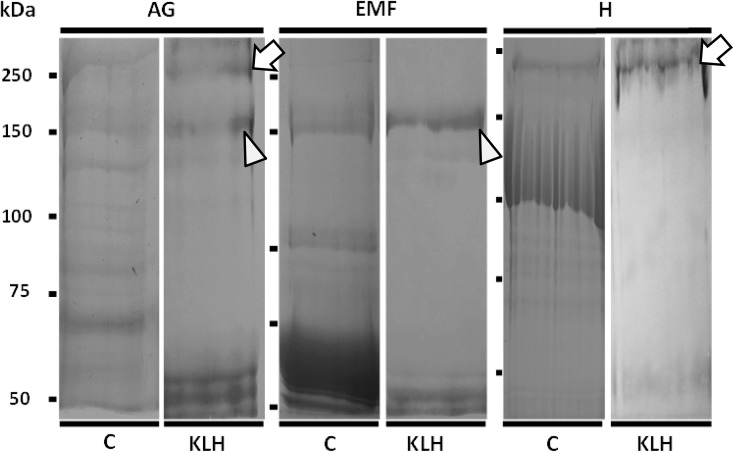
Hcl proteins in *Biomphalaria glabrata*. Protein samples of AG, EMF and hemolymph (H) were separated by 5–20% gradient SDS-PAGE and either stained with Coomassie (C) or probed with antiserum against Keyhole limpet hemocyanin (KLH). Electrophoresis was continued until the 50kDa marker reached the bottom of the gel to improve separation in the high molecular weight range. Coomassie staining shows the different protein compositions of the samples. Immunoblots show that the anti-KLH antiserum recognizes bands with the predicted molecular weight for Hcl proteins. Arrowheads indicate Hcl-1 (150kDa) in AG and EMF. Arrows indicate Hcl-2 (250 kDa) in the AG and H samples. The KLH antiserum also cross-reacted with smaller protein bands (~50 kDa) that were not analyzed in this study. Images shown are from separate gels, position of molecular weight markers in kDa is indicated with bars to the left of the images.

The Coomassie stained gel profiles indicate differences in the protein composition between the samples. Yet, both AG and EMF contain an α-KLH antiserum reactive protein band at 150 kDa (Fig 7), the predicted molecular weight of Hcl-1. The protein profiles of Hemolymph and AG of *B*. *glabrata* showed reactivity with α-KLH at 250 kDa, the predicted molecular weight for Hcl-2 (Fig 7). The α-KLH polyclonal antibodies also cross-reacted with several protein bands of ~50kDa that were not analyzed further in this study.

Consideration of combined peptide mass fingerprinting (PMF) and tandem MS data from the trypsin-digested 150kDa protein bands yielded three peptide sequences (two shared between both samples, one only from EMF) that led to positive identification of Hcl-1 (*p* <0.05). At a 1 Da precursor-ion tolerance, from 85 mass values recorded from AG and 73 from EMF, another eleven peptide masses from PMF matched *in silico* predicted tryptic peptide fragments of Hcl-1 (Fig 4; S5 Table). None of the peptides recovered matched the Hcl-2 sequence.

## Discussion

The red-pigmented blood of *B*. *glabrata* underscores the fact that snails of the family Planorbidae are unique among gastropods in that they have replaced the ancestral (blue) hemocyanin as an oxygen-carrier with secondarily evolved hemoglobin. Nonetheless, hemocyanin-like (cDNA and peptide) sequences as well as a large blood protein that resembles the characteristic ultrastructural shape of multimeric molluscan hemocyanin have been described from *B*. *glabrata* [16, 20, 21]. We conclude that these observations do not stem from a respiratory molluscan hemocyanin, but rather, find a basis in two novel hemocyanin-like genes, termed *hcl*-1 and *hcl*-2.

The large hemolymph protein of *B*. *glabrata* with a hemocyanin-like shape [16, 20] originates from the *hcl-2* sequence. An antiserum raised against the heterologous keyhole limpet hemocyanin (KLH) recognizes a *B*. *glabrata* hemolymph protein with same ~250 kDa molecular weight that is predicted from the amino acid sequence of Hcl-2. Additionally, the gene sequence of *hcl-2* encodes a protein that comprises six FU domains (A-F), two fewer than the eight FUs from bonafide molluscan hemocyanins. This configuration is consistent with unusual aspects of the ultrastructural shape of the hemocyanin-like blood protein of *B*. *glabrata*, resulting from the absence of the so-called internal collar complex that is formed by the terminal FU domains G and H of regular molluscan hemocyanins [17]. Kokkinopoulou et al. [20] provided structural evidence that *B*. *glabrata* may express this protein (putatively encoded by *hcl-2*) in pore cells (rhogocytes) of the mantle. The immune detection of a ~250kDa protein band also from AG may indicate that Hcl-2 is also expressed in this organ, additional to rhogocytes [20]. Alternatively, this signal may be due to cross-reaction with an unrelated protein or the blood circulation may cause a hemolymph protein as Hcl-2 to be present in the AG. Relatively small amounts of the hemocyanin-like protein are present in the hemolymph, compared to hemoglobin, such that it is less likely to function for oxygen-binding alongside with hemoglobin [16, 20]. It may be expressed for a different purpose, however, possibly related to the prophenoloxidase/tyrosinase activity of molluscan hemocyanins [16].

The *hcl-1* gene sequence characterized in this study was initially reported from EST data and hemocyanin-like peptides recovered by mass spectrometry from *B*. *glabrata* AG and EMF [16, 21]. Our immunoblot results with the anti-KLH antiserum and novel, more extensive mass spectrometry data show that *B*. *glabrata* Hcl-1 is expressed as a 150kDa protein, concordant with the predicted molecular weight of amino acid sequence of the predicted mature protein. The *hcl-1* sequence represents a novel type of hemocyanin-like gene that has 3 FU domains, is expressed only in the AG and is contributed to the EMF. Perhaps, after being replaced as respiratory pigment by the evolutionary development of hemoglobin, the ancestral hemocyanin genes of planorbid snails were repurposed to serve other functions, in analogy to functional diversification of duplicated genes [40–42]. In this analysis of these two hemocyanin-like genes of *B*. *glabrata*, we focus on an initial characterization of *hcl-1* toward exploration of the hypothesis that *hcl-1* may play a role in defense, reproduction and/or egg viability of *B*. *glabrata*.

Studies of other molluscs have revealed the general structure of hemocyanin genes to provide a frame of reference to track the evolution of hemocyanins [43–47]. Hemocyanin genes of cephalopods (*Octopus dofleini* & *Nautilus pompilius*; [43, 45], respectively), a bivalve (*Nucula nucleus*; [46]) and gastropods (e. g. *H*. *tuberculata*; [44]) share a characteristic feature; the exons that encode FUs and the downstream linker sequences are consistently connected by phase one introns. This shared feature was proposed to serve a regulatory function and/or to facilitate duplication of single functional unit domains [43, 47]. Both *hcl-1* and *hcl-2* genes from *B*. *glabrata* also feature these phase one introns associated with linker-encoding sequences. This suggests that these genes are in fact evolutionarily related to oxygen-binding molluscan hemocyanins. The number of introns in the FU-encoding regions of the *B*. *glabrata hcl* genes is greater per FU than that of other traditional molluscan hemocyanins that have at most three introns within a FU [43–46, 48]. Intron gain and loss in orthologous genes is believed to occur at higher frequencies during major evolutionary transitions [49]. If *hcl*-1 and *hcl*-2 represent functionally diversified ancestral hemocyanins, the gain of introns associated with each FU may have aided in shaping a putative novel function for *hcl* genes in *B*. *glabrata* upon the arrival of hemoglobin in planorbid snails, through provision of intron regulatory sequences.

Molluscan hemocyanins contain specific conserved features that distinguish them from other copper type 3 proteins. First, the organization of multiple FU domains provides a significant difference from single FU domain proteins of the same superfamily, such as tyrosinases and phenoloxidases [27]. Both *B*. *glabrata* Hcl proteins have linker sequences of 10–15 amino acids that connect multiple FU domains, similar to molluscan hemocyanins. Second, amino acid level comparison of single FUs showed that component FU domains of both Hcl proteins are most similar to those of other molluscan hemocyanins, more so than to tyrosinases (Fig 3). Despite these similarities with respiratory hemocyanins of other molluscs, differences in sequence and, in case of Hcl-1, site of synthesis and a developmentally-dependent expression in *B*. *glabrata* snails suggest that Hcl proteins may not function in the oxygen-carrying role of other molluscan hemocyanins. Both Hcl proteins contain a lesser number of FU domains, and these domains do not include the full complement of canonical histidines of the sequence motif that mediates copper binding to facilitate O_2_ binding [17]. In the case of Hcl-1, the presence of only 3 FUs may not permit Hcl-1 to assemble into a multimeric protein as afforded by the multidomain structure of seven or more FUs that characterize O_2_-transporting molluscan hemocyanins. Finally, one of the six conserved histidines from each of the copper-binding motifs of hemocyanin is absent in Hcl-1 sequences from all four *B*. *glabrata* strains tested (Fig 4). This may impair the ability of Hcl-1 protein to bind and transport oxygen. In summary, comparative analysis of these proteins suggests that Hcl-1 and Hcl-2 are related to molluscan hemocyanins, but also support the notion that the purpose of *B*. *glabrata* Hcl-1 is not oxygen transport.

The synthesis of Hcl-1 uniquely in the AG is suggestive of the putative functional role that Hcl-1 may have in *B*. *glabrata* (Fig 5). The AG is an organ associated with the reproductive tract and it also has a role in immunity. Prophenoloxidase (PO) activity contributes to formation of egg shells in many invertebrates including *B*. *glabrata* [26]. A 35 kDa enzyme with PO activity from the AG of reproductively mature *B*. *glabrata* is critical for egg production, likely through crosslinking of egg shell proteins [50]. As a copper type 3 protein, Hcl-1 may also contribute PO activity needed for egg production in *B*. *glabrata*. However, the deposition of the 35 kDa tyrosinase and Hcl-1 in the EMF [21] suggests that these proteins may contribute to maintenance of shell structure and/or carry out other functions, perhaps immune-related.

In adult *B*. *glabrata* snails, the AG produces lectins also in response to parasitic infection [51]. Thus, Hcl-1 may play a role as an innate immune factor; some copper type 3 proteins possess PO activity towards melanization of pathogens [52, 53]. At the same time, protein components in the EMF of egg masses of *B*. *glabrata* have been implicated in immune defense of developing snail embryos against microbes. RNAi knockdown of the EMF component lipopolysaccharide-binding protein and bactericidal/permeability-increasing protein (LBP/BPI) from *B*. *glabrata* reduced fitness by lowering egg number per egg mass, and rendering egg masses highly susceptible to degradation by oomycetes [54]. Hcl-1 proteins are provided by adult *B*. *glabrata* to the perivitelline fluid surrounding developing embryos because the embryos themselves do not express Hcl-1 (Fig 6), this may be an example of vertical transfer of immunity in *B*. *glabrata*, providing protection against microbes to increase fitness of embryos. Alternatively, Hcl-1 may provide a different type of protection by facilitating darker pigmentation through enzymatic production of melanin. Melanin pigment protects against UV radiation [55, 56] and may increase tolerance of *B*. *glabrata* embryos against exposure to sunlight, augmenting egg survival in the environment.

Another alternative possibility is that Hcl-1 functions similar to arthropod hexamerins, which are hemocyanin-derived proteins [57–59]. Hexamerins function in maintenance of arthropod physiology as a source of nutrition by providing amino acids to non-feeding larvae [60–62]. Hexamerins also lack at least one of the histidines of the copper-binding motifs that mediate O_2_ binding, rendering them non-functional for oxygen transport [59]. Hemocyanins in arthropods and molluscs resulted from convergent evolution and are only distantly related [57]; nevertheless, Hcl-1 may serve a similar function to hexamerins as a source of nutrition for developing *B*. *glabrata* inside the egg masses.

Finally, to our knowledge, Hcl-1 has been identified only in *B*. *glabrata*. Due to the occurrence of hemoglobin, it may have evolved from an ancestral hemocyanin after relaxed evolutionary pressure to function as the oxygen transporter for the family Planorbidae [16]. It remains to be determined if the development of Hcl-1 is unique to planorbid snails or if Hcl-1 diversified from hemocyanin in their ancestors such that it is also present in other gastropod families that do employ hemocyanin for oxygen transport, such as Physidae and Lymnaeidae, which are air-breathing freshwater snails in the clade Hygrophila together with the Planorbidae [63]. In that case, Hcl-1 may represent a novel category of copper type 3 related proteins not previously recognized in gastropods. In the case of *B*. *glabrata*, we hypothesize that Hcl-1 supports successful development of embryos either by O_2_ transport, production of melanin for pigmentation/defense, serving as a source of nutrition for eggs, or all of these. Further studies are needed to investigate the distribution of this protein among gastropod snails, to better understand the function of Hcl-1 and to explore Hcl-1 as a potential target for snail control in aid of efforts to reduce transmission of schistosomiasis.

## Supporting Information

S1 Fig *Biomphalaria glabrata* Hcl-2, alignment of functional unit domains with copper type 3 enzymes. The Hcl-2 FU domains (Hcl-2_1 through Hcl-2_6) were aligned with a tyrosinase sequence of the bivalve *Crassostrea gigas* (CrTyr) and four predicted tyrosinases of *B*. *glabrata* (Bgtyr). Amino acids relevant for the copper binding motif are highlighted in yellow; amino acid replacements are shown in red. Initial alignment was done with ClustalW and checked visually using MEGA 6.
(EPS)

S1 Table Hcl-1 primers used for sequencing. Asterisks identify primers used for RT-PCR of tissue-specific (*) and age-dependent (**) experiments (v = a/c/g; n = a/t/c/g).
(DOCX)

S2 Table Features of *hcl-1* introns. The *hcl-1* gene contains 23 exons. The FUs have a greater number of introns when compared to other molluscan hemocyanins. An asterisks (*) identifies introns between FUs that contain the conserved phase one codon interruption characteristic of in molluscan hemocyanins.
(DOCX)

S3 Table Features of *hcl-2* introns. The *hcl-2* gene contains 34 exons. The FUs have a greater number of introns when compared to other molluscan hemocyanins. Introns between FUs contained the conserved phase one codon interruption previously recognized in molluscan hemocyanins (highlighted by asterisks).
(EPS)

S4 Table Synonymous mutations among Hcl-1 transcripts of four *B*. *glabrata* strains. The numbers refer to locations indicated in the alignment of Hcl-1 sequences among four *B*. *glabrata* strains (Fig 4).
(EPS)

S5 Table Hcl-1 peptides recovered from Peptide Mass Fingerprinting (PMF) and MS/MS analysis of 150 kDa protein band from AG and EMF samples of *B*. *glabrata*. Consideration of combined peptide mass fingerprinting and tandem MS data (PMF/MS) yielded three peptide sequences that led to positive identification of Hcl-1 (*p* <0.05). At a 1 Da precursor-ion tolerance, another additional eleven peptide masses (PMF) matched in silico predicted tryptic peptide fragments of Hcl-1. FU = Functional unit of Hcl-1 (A,B and H) where peptide sequences are located in Fig 4, AG = Albumen gland, EMF = Egg mass fluid.
(DOCX)
