# Supplementary material for: The Planorbid Snail Biomphalaria glabrata Expresses a Hemocyanin-Like Sequence in the Albumen Gland
Source: PLoS One. 2016 Dec 30;11(12):e0168665. doi: 10.1371/journal.pone.0168665 (PMC5201427; doi:10.1371/journal.pone.0168665)
Supplement: S1 Table — Asterisks identify primers used for RT-PCR of tissue-specific (*) and age-dependent (**) experiments (v = a/c/g; n = a/t/c/g). (DOCX) [file pone.0168665.s002.docx]

**Supplemental table 1: Hcl-1 primers used for sequencing.** Asterisks identify primers used for RT-PCR of tissue-specific (*) and age-dependent (**) experiments (v = a/c/g; n = a/t/c/g).

| Hcl-1 primers | 5'-3' |
| --- | --- |
| 5'UTR | TTCAGTACAGCCCATGGACTGGTTTAAAG |
| 2R | GCAAGATACAGCCTGTGCC |
| 2F | TGCCGAGGCCTGATTCTTAGAAAAG |
| 5R | AGTCAAAGACGGTCTTGTCAGTCATG |
| 5F | ATCCCAACTATTGCCAGTTTGTGGTG |
| 9F** | TGATCCAATCTTTTACATTCATCAC |
| 12R | ATATGGACCAGATCTTATC |
| 12F | TGATCCAATCTTTTACATTCATCAC |
| 13R** | AAGTTGTCAGCGCTAGTCTCA |
| 15R | AAGTTGTCAGCGCTAGTCTCA |
| 15F | AGAAAGAACATCCAAGAGCTGACAG |
| 20R | ACTCAAAGACCTGGAGAC |
| 21R* | TTGACGTTACTGACGCCATGA |
| 23R* | AAGTCAAGTTTGTTACCGACGACG |
| PolyTanchor | TTTTTTTTTTTTTTTTTTVN |
