# Supplementary material for: The Planorbid Snail Biomphalaria glabrata Expresses a Hemocyanin-Like Sequence in the Albumen Gland
Source: PLoS One. 2016 Dec 30;11(12):e0168665. doi: 10.1371/journal.pone.0168665 (PMC5201427; doi:10.1371/journal.pone.0168665)
Supplement: S2 Table — The hcl-1 gene contains 23 exons. The FUs have a greater number of introns when compared to other molluscan hemocyanins. An asterisks (*) identifies introns between FUs that contain the conserved phase one codon interruption characteristic of in molluscan hemocyanins. (DOCX) [file pone.0168665.s003.docx]

**Supplemental Table 2: Structure of *hcl-1* intron phases**. The hcl-1 gene contains 23 exons. The FUs have a greater number of introns when compared to other molluscan hemocyanins. An asterisks (*) identifies introns between FUs that contain the conserved phase one codon interruption characteristic of in molluscan hemocyanins.

| **Hcl-1** | | | |
| --- | --- | --- | --- |
| **Intron #** | **Intron ‘5-3’** | **Intron phase** | **FU** |
| **1** | **GT…CAG** | **-** | **sp** |
| **2** | **GT…CAG** | **1*** | **A** |
| **3** | **GT…GAG** | **0** | **A** |
| **4** | **GT…CCG** | **1** | **A** |
| **5** | **GT…TAG** | **2** | **A** |
| **6** | **GT…GAG** | **0** | **A** |
| **7** | **GT…AGC** | **1*** | **B** |
| **8** | **GT…CAG** | **0** | **B** |
| **9** | **GT…TAG** | **0** | **B** |
| **10** | **GT…GAG** | **2** | **B** |
| **11** | **GT…CAG** | **0** | **B** |
| **12** | **GT…CAG** | **2** | **B** |
| **13** | **GT…TCA** | **1** | **B** |
| **14** | **GT…CAG** | **2** | **B** |
| **15** | **GT…CAG** | **1*** | **H** |
| **16** | **GT…CAG** | **1** | **H** |
| **17** | **GT…TAG** | **0** | **H** |
| **18** | **GT…TAG** | **0** | **H** |
| **19** | **GT…CAG** | **0** | **H** |
| **20** | **GT…TAG** | **0** | **H** |
| **21** | **GT…CAG** | **0** | **H** |
| **22** | **GT…CAG** | **0** | **H** |
