# Supplementary material for: The Planorbid Snail Biomphalaria glabrata Expresses a Hemocyanin-Like Sequence in the Albumen Gland
Source: PLoS One. 2016 Dec 30;11(12):e0168665. doi: 10.1371/journal.pone.0168665 (PMC5201427; doi:10.1371/journal.pone.0168665)
Supplement: S5 Table — Consideration of combined peptide mass fingerprinting and tandem MS data (PMF/MS) yielded three peptide sequences that led to positive identification of Hcl-1 (p <0.05). At a 1 Da precursor-ion tolerance, another additional eleven peptide masses (PMF) matched in silico predicted tryptic peptide fragments of Hcl-1. FU = Functional unit of Hcl-1 (A,B and H) where peptide sequences are located in Fig 4, AG = Albumen gland, EMF = Egg mass fluid. (DOCX) [file pone.0168665.s006.docx]

**Supplemental table 5. Hcl-1 peptides recovered from Peptide Mass Fingerprinting (PMF) and MS/MS analysis of 150 kDa protein band from AG and EMF samples of *B. glabrata.*** Consideration of combined peptide mass fingerprinting and tandem MS data (PMF/MS) yielded three peptide sequences that led to positive identification of Hcl-1 (*p* <0.05). At a 1 Da precursor-ion tolerance, another additional eleven peptide masses (PMF) matched in silico predicted tryptic peptide fragments of Hcl-1. FU= Functional unit of Hcl-1 (A,B and H) where peptide sequences are located in Fig.4, AG= Albumen gland, EMF= Egg mass fluid. (lines 752-759)

|  |  |  |  |
| --- | --- | --- | --- |
| Sample | Peptide Sequence | Method | FU |
| EMF | SYSYIASFYGYPTR | PMF | A |
| AG, EMF | IYEVYEALYR | PMF | A |
| EMF | DDNPFPNTR | PMF | A |
| AG, EMF | RPYYHDITK | PMF | A |
| EMF | QDVDLLTEDEMNALR | PMF | B |
| AG, EMF | GLPSFPHWHR | PMF | B |
| EMF | ANPFYDAAIEFLR | PMF | B |
| EMF | AEIHATLHTGER | PMF/MS | B |
| EMF | LQSETSADNFENIAGFHGAPNR | PMF | H |
| AG, EMF | LLTVQFEQALSR | PMF/MS | H |
| AG, EMF | LFSDPEDNPFYR | PMF/MS | H |
| EMF | HVDSSMEPFR | PMF | H |
| EMF | ENSLPGLVSDHR | PMF | H |
| EMF | TGTYDGSSSSEK | PMF | H |
